# An unusual case of bilateral renal enlargement due to primary renal lymphoma

**DOI:** 10.4103/0971-4065.78081

**Published:** 2011

**Authors:** S. C. Dash, K. Purohit, S. K. Mohanty, A. K. Dinda

**Affiliations:** Department of Nephrology, Kalinga Institute of Medical Sciences, Bhubaneswar, India; 1 Department of Pathology, Kalinga Institute of Medical Sciences, Bhubaneswar, India; 2 Department of Pediatric Surgery, Kalinga Institute of Medical Sciences, Bhubaneswar, India; 3 Department of Pathology, All India Institute of Medical Sciences, New Delhi, India

**Keywords:** Extranodal lymphoma, normal renal function, symmetrical renal enlargement

## Abstract

Primary renal lymphoma is an uncommon variant of extranodal non-Hodgkin’s lymphoma. Manifestations are usually nonspecific hematuria, fever, flank pain, and renal insufficiency. Pathological data are scanty; few reports indicate it has a very poor prognosis. We describe a child with bilateral symmetrically palpable kidneys, low-grade pyrexia, and arthralgia. Clinically, diagnosis was missed partly due to the fact that bilateral large renal tumors commonly produce asymmetric renal swelling, renal dysfunction, and hematuria which were absent in this case and partly due to rarity of the condition. However, radiological investigations combined with renal histology helped in establishing diagnosis in the present case.

## Introduction

Primary renal lymphoma (PRL) is rare. Kidneys are also an uncommon primary extranodal lymphoma (ENL) location.[1,2] Middle aged and elderly people are mostly affected with an incidence of 0.7% among all cases of ENL.[1–4] Pathogenesis is unclear and treatment of PRL is ambiguous. Diagnosis is often delayed due to nonspecific nature of manifestations. Early detection therefore depends on combination of computed tomographic scan and examination of adequate biopsy material. We describe a child who presented to pediatric and then to nephrology units with low-grade pyrexia, severe loss of weight, and bilateral kidney swelling.

## Case Report

A seven-year old girl presented to emergency room with 5 months history of intermittent fever, joint pain, severe anemia, and distended abdomen. She was treated outside by alternative medicine (‘naturopathy’) based on severe diet restrictions consisting of few pieces of bread and vegetables for several months. She was also misdiagnosed, hence treated wrongly as a case of congestive heart failure. Bilateral renal enlargement was mistaken for congested hepatosplenomegaly. No proper evaluation for abdominal distention was made at this time.

**Figure 1:**
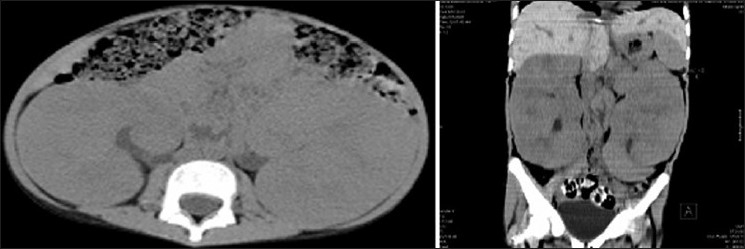
CT scan showing bilateral diffuse renal enlargement

At admission to our hospital, physical examination revealed an emaciated child with severe anemia and bilateral palpable kidneys. Clinically they were symmetrical, non-cystic, and non-tender. Routine laboratory evaluation showed a blood urea nitrogen level of 16 mg/dl, serum creatinine 0.9 mg/dl, and uric acid was 7.8 mg/dl. Creatinine clearance was 52 ml per minute. Her hemoglobin was 3.2 g/dl, total leukocyte count 7900/mm^3^, and platelet count 2.53 × 10^4^/mm^3^. Peripheral smear showed moderate anisocytosis with macrocytes, polychromatic cells, normocytes, and few microcytic normochromic cells suggesting hemolytic anemia. Reticulocyte count was 4.5 percent. Serum calcium was 10.2 mg/dl, phosphorous 3.1 mg/dl, and uric acid 9.8 mg/dl. Liver function tests were normal. The erythrocyte sedimentation rate was 38 mm/h and C-reactive protein was 1.4 mg/dl. Tuberculin skin test and serological assays for EB virus and virus were negative. Serum compliment and quantitative estimation of immuno-globulins were non-contributory. Urinalysis revealed trace protein but no active sediments. Repeated urine cytology revealed no malignant cells. Ultrasound of abdomen revealed marked bilateral renal enlargement with homogenous cortical echogenicity. The kidneys measured 17.9 × 6.8 × 7.1 and 16.6 × 6.8 × 7.1 cm. There was mild hepatomegaly without evidence of any focal lesion. Intrahepatic vascular and biliary radicles were normal. A plain computed tomography (CT) scan of abdomen confirmed bilateral diffused renal enlargement with decreased attenuation suggestive of diffuse infiltrative disease. There was mild hepatomegaly but no evidence of hydronephrosis or intra-abdominal lymphadenopathy [Figure 1]. Chest X-ray showed normal lung parenchyma and a normal size heart without any mediastinal lymphadenopathy, which was confirmed by CT scan.

**Figure 2:**
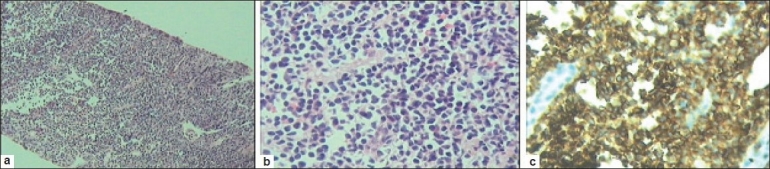
(a,b) Needle biopsy of kidney low and high magnification showing tumor infiltration confined to interstitium. (c) Immunohistochemistry with CD45 marker study

Percutaneous renal biopsy showed extensive infiltration of interstitium by monomorphic neoplastic cells, some of which were round with round nuclei and scanty cytoplasm. Tumor cells were arranged in a tubular pattern at places. Tumor infiltration was found confined to interstitium without involving glomeruli or tubule [Figure 2a and b]. CD45 showed surface positivity in all cells [Figure 2c]. Bone marrow biopsy was unremarkable, as were the head and chest CT scans. In view of angulation of all nucleoli, mucosa-associated lymphoid tissue (maltoma), a form of PRL was entertained.

She was put on combination chemotherapy consisting cyclophosphamide, intravenous vincristine 2 mg/M^2^and shifted to a medical oncology unit where she was started on combination chemotherapy with CHOP (cyclophosphamide, doxorubicin, vincristine, and prednisolone). She developed serious leucopenia (total leukocyte count of 900/cmm) on sixth postchemotherapy day, needing temporary suspension of therapy and institution of broad spectrum antibiotics.

## Discussion

Bilateral symmetrical renal enlargement can be due to bilateral hydronephrosis, cystic diseases, or due to malignant or benign infiltrative diseases. Rarely, it can occur in bilateral renal vein thrombosis. However, these conditions were easily ruled out on the basis of clinical features, ultrasonogram, and CT scan. CT scan showed diffuse enlargement of kidneys. Wilms’ tumor is seen in about 5% of children involving both kidneys; generally renal enlargement is asymmetric and imaging techniques would show tumor attenuation different from renal parenchyma. Other renal tumors were similarly ruled out.

Acute phase reactant levels were normal and immunological parameters were negative, suggesting malignancy as the diagnosis. Normal blood urea nitrogen and creatinine levels indicating absence of significant renal dysfunction in this case was rather surprising finding in view of bilateral diffuse extensive involvement of both kidneys. However, her creatinine clearance was decreased to 52 ml per minute. Hydronephrosis due to obstruction of renal pedicles or pelvis by lymphnode enlargement was ruled out by ultrasonogram.

As has been stated earlier, PRL is a rare disease hence poses difficulty in diagnosis.[5,6] Less than 100 cases of PRL have been reported in the literature, many of whom were not investigated by imaging and bone marrow biopsy to rule out extra renal disease.[7] On the other hand, there is considerable doubt among pathologists over development of lymphoma, because kidneys do not have identifiable lymphatic channels. Some pathologists tend to think PRL is a disseminated malignancy, because in 10 to 20% of cases lymphoma affects both kidneys,[3,6] as was in the present case. Mucosa-associated lymphoid tissue renal lymphoma is probably the underlying mechanism.[8]

The imaging morphology of PRL can be variable from unilateral to bilateral single or multiple masses, diffuse parenchymal infiltration.[3,8] Contrast-enhanced CT scan is ideal test is useful, but was avoided in her case for potential risk of contrast-induced nephropathy, as her glomerular filtration rate was low (52 ml/mt) and due to unavailability of consent from her parents. Diagnosis was made on the basis of histology and immunohistochemistry. Fine needle aspiration or core renal biopsy clearly is the best method to establish diagnosis with high specificity and sensitivity.

Treatment of PRL remains controversial because pathogenetic mechanisms are uncertain. Role of radiation as a therapy in treatment of bilateral diffuse lymphoma infiltration is not established. It is equally unclear if it should be only treated by chemotherapy or in combination with radiation. Lack of therapeutic trials in literature has obviously compelled clinicians to adopt a practical approach and treat according to histological grade. As it has been established that most cases of PRL are high-grade lymphoma mainly of B-cell phenotype, they are treated by CHOP or other chemotherapy regimen with or without radiotherapy.[3,7] However, results are unsatisfactory due to rapid progression of disease and treatment resistance. Recently, rituximab has been added to CHOP regime which is expected to improve the outcome of PRL of B-cell origin; although, therapy of PRL will remain empirical in the absence of large multicenter or multinational trials.

In conclusion, PRL should be suspected if there is bilateral enlargement of the kidneys. Modern imaging techniques and renal histology have a central role in establishing the diagnosis.
